# Chronic Pain Patients’ Gaze Patterns toward Pain-Related Information: Comparison between Pictorial and Linguistic Stimuli

**DOI:** 10.3390/medicina55090530

**Published:** 2019-08-25

**Authors:** Jieun Lee, Jaewon Beom, Seoyun Choi, Seulgi Lee Amy Wachholtz, Jang-Han Lee

**Affiliations:** 1 Department of Psychology, Chung-Ang University, Seoul 06974, Korea; 2 Department of Physical Medicine and Rehabilitation, Chung-Ang University Hospital, Chung-Ang University College of Medicine, Seoul 06973, Korea; 3 Department of Psychology, University of Colorado-Denver, Denver, CO 80204, USA

**Keywords:** attentional preference, linguistic, visual stimuli, chronic pain

## Abstract

*Background and Objectives:* The attentional bias and information processing model explained that individuals who interpret pain stimuli as threatening may increase their attention toward pain-related information. Previous eye tracking studies found pain attentional bias among individuals with chronic pain; however, those studies investigated this phenomenon by using only one stimulus modality. Therefore, the present study investigated attentional engagement to pain-related information and the role of pain catastrophizing on pain attentional engagement to pain-related stimuli among chronic pain patients by utilizing both linguistic and visual stimulus. *Materials and Methods:* Forty chronic pain patients were recruited from the rehabilitation center, the back pain clinic, and the rheumatology department of Chung-Ang University Hospital in Seoul, Korea. Patients observed pictures of faces and words displaying pain, presented simultaneously with neutral expressions, while their eye movements were measured using the eye tracking system. A *t*-test and ANOVA were conducted to compare stimulus pairs for the total gaze duration. *Results:* Results revealed that chronic pain patients demonstrated attentional preference toward pain words but not for pain faces. An ANOVA with bias scores was conducted to investigate the role of pain catastrophizing on attentional patterns. Results indicated that chronic pain patients with high pain catastrophizing scores gazed significantly longer at pain- and anger-related words than neutral words compared to those with low pain catastrophizing scores. The same patterns were not observed for the facial expression stimulus pairs. *Conclusions:* The results of the present study revealed attentional preference toward pain-related words and the significant role of pain catastrophizing on pain attentional engagement to pain-related words. However, different patterns were observed between linguistic and visual stimuli. Clinical implications related to use in pain treatment and future research suggestions are discussed.

## 1. Introduction

Attentional bias toward pain-related information is well established [1]. However, the evidence for attentional bias toward pain stimuli has not always been supported and authors argued that the inconsistency in results is due to differences in the methodologies [1,2]. These meta-analytic studies found consistent evidence for attentional engagement toward pain-related information for maintained attention at the supraliminal level (i.e., presentation time longer than 1250 ms) but inconsistent results at the subliminal level (i.e., presentation time shorter than 500 ms). These results indicated a need for researchers to consider a stimulus presentation time that is longer than 1250 ms in order to examine maintained attention to pain-related stimuli. Furthermore, to address limitations associated with the visual-probe task (i.e., attentional bias to specific stimuli can only be observed at certain specified time points), an eye tracker was used to observe attentional patterns toward pain-related stimuli continuously across time in recent attentional bias studies.

Previous studies utilizing eye tracking methods [3,4,5] found that individuals with chronic pain have a tendency to gravitate toward pain stimuli in their attentional process. However, literature indicated that attentional bias is “a complex process”, which includes not only attentional engagement toward threatening stimuli but also attentional avoidance from threatening stimuli [6]. Previous studies [6,7] found attentional avoidance from threat-related information when facing real threatening and painful conditions (e.g., operation, dental treatment). This result indicates that when the degree of threat is high (i.e., situations where individuals anticipate high levels of acute pain), individuals may turn their attention away from threatening stimuli in order to manage anxiety. When a threat becomes chronic, chronic pain patients demonstrated difficulty in disengaging their attention from pain stimuli [3,4,5].

Maintaining attention toward pain-related information shown in chronic pain patients [3,4,5] may be an attentional process similar to the rumination process observed in depressed individuals. This implies that repetitive negative thoughts related to pain increase and maintain the attention of pain suffers to pain-related information [1]. Pain catastrophizing is defined as cognitive and emotional responses (i.e., rumination, exaggeration, and helplessness) to actual or anticipated pain. Pain catastrophizing is known to be one of the important psychological factors associated with frequency and severity of chronic pain [8,9,10]. Previous eye tracking studies [3,4] implied attentional engagement toward pain-related information could be influenced by pain catastrophizing, however, those studies were conducted with a non-clinical population. The current study investigated the role of pain catastrophizing on maintained attention toward pain-related information in a clinical population in order to confirm previous results obtained from non-clinical sample studies.

Meta-analytic studies mentioned above suggested that one of the reasons why attentional bias studies demonstrated inconsistent results was due to different stimulus types [1,2]. People may respond to visual and linguistic stimuli differently. According to the dual-coding theory, visual and linguistic information are processed through distinct channels in the human brain [11]. The results of previous studies [12,13,14] support the notion suggested by the dual-coding theory (i.e., differences in the processing between visual and verbal information). Those studies found not only functional differences but also anatomical differences in processing visual and linguistic stimuli in human brain. Although previous studies found evidence for attentional engagement toward pain-related linguistic stimuli [1,2] and attentional engagement toward pain-expression pictures [3,4,5] among chronic pain individuals, similarities and differences in the attentional engagement of chronic pain individuals toward different pain stimulus modalities have rarely been compared using eye tracking methodology. Studies, such as ours, that directly compare different pain stimulus modalities as part of investigating the pain attentional bias of chronic pain patients will provide valuable information to develop the most effective psychological intervention for chronic pain patients.

In summary, the present study examined how the attentional engagement toward pain-related information differs depending on different stimulus modalities (word vs. facial expression) and a psychological factor, particularly pain catastrophizing levels. Moreover, to investigate pain-specific attentional patterns, anger-related information pairs (anger vs. neutral stimuli) were included and compared with pain-related stimuli pairs. Inconsistent results in previous attentional bias studies among individuals with chronic pain might have occurred due to a diversity of chronic pain conditions, therefore the current study only recruited patients who were diagnosed with musculoskeletal disorders. Based on previous studies, the present study proposed that (1) chronic pain patients would demonstrate attentional engagement toward pain-related information regardless of types of stimuli presented (i.e., word and pictorial stimuli); (2) attentional engagement toward pain-related information would be maintained across 3000 ms for both pain-related word and pictorial stimuli; and (3) attentional engagement toward pain-related information would be influenced by pain catastrophizing levels for both pain-related word and pictorial stimuli.

## 2. Materials and Methods

### 2.1. Participants

We conducted the power analysis of repeated measure ANOVA with within-subjects using G*Power 3.1 program and 28 participants (effect size = 0.25, type I error = 0.05, power of test = 0.80) were the minimum sample size. To account for a possibility of a minimum 10% dropout rate, we increased the sample size to 40. Forty patients with chronic pain that was diagnosed by medical doctors were recruited from the rehabilitation center, the back pain clinic, and the department of rheumatology in Chung-Ang University Hospital in Seoul, Korea. Inclusion criteria were: (1) aged 18 years or older; (2) having no cognitive disability; (3) having no difficulty in understanding and completing the survey; (4) having no eye diseases such as glaucoma and cataract; (5) experiencing chronic pain for more than 3 months; and (6) experiencing musculoskeletal pain as a major source of pain. After medical doctors referred patients to research assistants, the aims and design of the study were explained to patients and informed consent obtained from those who agreed to participate in the present study. This study was approved by the Institutional Review Board of Chung-Ang University Hospital and conforms to the Ethical Guidelines of the 1975 Declaration of Helsinki (approval number IRB#: 1813-002-313, approved on 28 November 2018).

### 2.2. Measures

Participants completed a questionnaire that contained demographic questions (i.e., age, gender, smoking and drinking) and pain-related questions (i.e., current pain level, levels of pain in the past month, and levels of pain in past 3 months). Regarding pain-related questions, the participants rated their pain on an 11-point numeric rating scale (NRS). In addition to the brief survey regarding demographic and pain-related questions, the following measures were administered.

A Korean-language version of the pain catastrophizing scale (PCS) has been translated and standardized in Korean population [15]. The original PCS was developed by Sullivan et al. [16]. The PCS has 13 items that assess three components of pain catastrophizing: rumination (e.g., “I can’t seem to keep it out of my mind”), magnification (e.g., “I wonder whether something serious may happen”), and helplessness (e.g., “There is nothing I can do to reduce the intensity of pain”). Participants were asked to rate their responses on a five-point Likert scale ranging from 0 (not at all) to 4 (always). Cronbach’s alpha of K-PCS was 0.93 [15] and internal consistency of this study was 0.91.

The pain disability index (PDI) that was originally developed by Pollard [17] and the present study utilized the Korean-language version of the PDI which was translated by Hong [18]. PDI is a questionnaire that measures the degree of subjective disruption experienced due to chronic pain in seven different domains of life (e.g., home, social, recreational, occupational, sexual life, self-management, and life-support). For each life domain, participants were asked to provide disability ratings on 11-point scales from 0 (no disability) to 10 (total disability). The higher score represents pain interfering more with life. Cronbach’s alpha was 0.87 [18] and internal consistency of this study was 0.91.

### 2.3. Stimulus Materials

#### 2.3.1. Photo Stimulus

As an experimental stimulus, photos displaying facial expressions were obtained by the Korea University Collection (KUFEC) [19] that contained different facial expressions such as anger, happiness, and neutral. Since facial expressions displaying pain were not included in the KUFEC, 32 photos displaying pained expressions were obtained from the previous study [3]. Two pilot studies were conducted to validate photos displaying pained expressions. In pilot studies, 15 participants (five male graduate students, 10 female graduate students) rated the extent manufactured photos are related to pain expression and valence of each photo on a 7-point scale. Based on the results of pilot studies, eight photos (four male, four female) were selected. The mean of rating for pained facial expression was M = 5.67, SD = 0.51 and the mean of rating for valence of pained facial expressions was M = 4.79, SD = 0.30.

Regarding photos displaying anger-related and neutral expression, pictures from the KUFEC corresponding to the selected painful faces were selected. A total of 24 pictures were used in this study (eight pain, eight anger, eight neutral).

#### 2.3.2. Word Stimulus

As another visual experimental stimulus, words describing different emotions including the state of experiencing pain (8 sensory pain words, 8 anger-related words, and 8 neutral words) were utilized in the present study. Total 24 sensory pain words were extracted from the previous studies [20,21]. The present study only included sensory pain words because previous studies including a meta-analysis study indicated that attentional bias was reliably established only to sensory pain words [2,22]. Two pilot studies (9 pain patients, 30 undergraduate and graduate students) were performed to select and validate sensory pain words. Participants rated the degree each word is related to sensory pain expression and rated valence for each word on a 7-point scale. As a result, 8 sensory pain words were selected (Degree each word is related to pain expression: M = 5.26, SD = 0.25; valence of pain words: M = 5.11, SD = 0.33).

Eight anger-related and neutral words were selected from the National Korean Language Institute and anger-related and neutral words were comparable in terms of their word lengths with pain words. All word stimuli were noun forms.

#### 2.3.3. Experimental Design

The paradigm of the experiment in the present study was based on previous studies [3,23,24,25]. Pairs of photos and words displaying different emotions were presented side by side on the gray background. The eye movements during viewing the stimuli were measured by the Tobii TX300 eye tracker system, which included an LCD 22-inch monitor screen and a camera attached to the bottom of the screen. The word and the picture stimuli were presented in the following order: a central fixation point (1000 ms), picture and word stimuli (3000 ms), and the blank screen (1000 ms). After sitting on a chair, the participants were asked to hold their head still to minimize head movement and view the stimuli through a monitor which was located 60 cm away from the chair. All words and photos were presented with a resolution of 1920 × 1080 pixels and the viewing angle was 38°.

In terms of stimuli, in total 32 picture pairs and word pairs (i.e., 16 pairs of ‘pain–neutral’, 16 pairs of ‘anger–neutral’) were shown. Each photo and word appeared equally on the left and right side to control the position bias (i.e., the habit of reading from the left to the right) [3]. To prevent the effect of learning influencing the gaze behaviors of the participants, the presentation sequence of picture and word stimuli was randomized for each participant. The Tobii TX300 eye tracker system defined fixation as the amount of time participants did not deviate their gaze from a defined area of interest (AOI). AOI for the photo and word stimuli was established based on the previous studies [3,21]. To assess the attentional engagement to different stimuli, the total gaze duration for each stimulus (i.e., the averages of total fixation durations within an AOI during the specified time) was calculated. The 3000 ms presentation time was selected to observe maintained attention [3].

### 2.4. Procedure

After arrival at the laboratory, participants completed the written consent. Prior to the experiment, participants were told to view photos and words on the screen as if they were watching television or reading a magazine. During the experiment, research assistants instructed participants to minimize their head motion and avoid talking for the entire experiment, which lasted 25 min. After the experiment, participants completed the questionnaires. The debriefing of the present study and monetary reward were provided after participants completed the questionnaire. Before each participant left the laboratory, handouts containing information regarding some relaxation techniques (i.e., diaphragmatic breathing, guided imagery) were provided and participants were assisted in learning and practicing relaxation techniques by two research assistants if participants expressed their interest in practicing those techniques.

### 2.5. Statistical Analysis

Correlational analyses among demographic information, pain-related outcomes, psychological factors, and total fixation durations toward pain, anger, neutral linguistic and pictorial stimuli were conducted. Pain and anger stimuli were compared with neutral stimuli by performing *t*-tests, and similarities and differences between the pictorial stimuli and linguistic stimuli were examined. Repeated measurement analyses of variance (ANOVAs) with the within-factors ‘time’ (0–500 ms vs. 500–1000 ms vs. 1000–1500 ms vs. 1500–2000 ms vs. 2000–2500 ms vs. 2500–3000 ms) and ‘stimulus type’ (pain vs. neutral, anger vs. neutral) were conducted for pictorial stimuli and linguistic stimuli to investigate the time-course of matained attentional patterns toward pain and anger stimuli. Lastly, ANOVAs were performed to test the role of pain catastrophizing on pain attentional bias for the pictorial and linguistic stimuli separately.

## 3. Results

### 3.1. Participant Characteristics

Table 1 shows descriptive statistics for the demographic characteristics of the participants (i.e., age, gender, smoking, drinking), pain-related variables (i.e., usage of pain medications, chronic pain durations, current and 3-month pain intensity, pain days per month, pain disability index, chronic pain locations), and pain catastrophizing.

We conducted inter-correlation analyses among demographic information (i.e., age, gender), pain-related variables (i.e., pain duration, pain frequency, pain disability index), pain catastrophizing, and gaze duration variables (i.e., total gaze duration toward pain stimulus in pain–neutral facial expression pairs and anger stimulus in anger–neutral facial expression pairs, total gaze duration toward pain words in pain–neutral word pairs and anger words in anger–neutral word pairs). The results revealed no significant correlations between demographic variables (i.e., age, gender) and other variables including pain catastrophizing, pain-related variables, and gaze duration variables; except age was positively correlated with pain duration (*r* = 0.347, *p* = 0.028). Pain catastrophizing was positively correlated with pain duration (*r* = 0.383, *p* = 0.015), total gaze duration for pain-related words in pain–neutral pairs (*r* = 0.376, *p* = 0.018) and total gaze duration for anger-related words in anger–neutral pairs (*r* = 0.381, *p* = 0.017). These results indicated that participants who reported high levels of pain catastrophizing were more likely to report longer pain duration and display higher total gaze duration for pain-related words and anger-related words compared to participants with low levels of pain catastrophizing.

ANOVA and chi-squared tests were performed to determine whether there were significant differences in demographic information and pain-related variables between high catastrophizing group and low catastrophizing group. Table 2 shows that no significant differences were found except for pain catastrophizing scores.

### 3.2. Total Gaze Patterns toward Different Pairs: Comparison between Pictorial and Linguistic Stimuli

As shown in Table 3, opposite attentional patterns were observed between facial expressions and word stimuli. For facial expressions, attentional preferences toward neutral stimuli in pain–neutral and anger–neutral pairs were observed, while the reverse was seen in word stimuli with preferential attentional patterns toward threat-related stimuli in pain–neutral and anger–neutral pairs.

In order to further investigate any potential interactions between stimulus type (word vs. picture) and expression type (pain vs. neutral, anger vs. neutral), ANOVA was conducted. Table 4 revealed that a significant result was found only for the interaction effect (*F* (1, 152) = 7.018, *p* < 0.001, *η*^2^ = 0.044) for pain and neutral pairs. Regarding anger and neutral pairs, a significant main effect for stimulus type (*F* (1, 152) = 5.427, *p* < 0.05, *η*^2^ = 0.034) and an interaction effect (*F* (1, 152) = 4.184, *p* < 0.05, *η*^2^ = 0.027) were found. 

**Figure 1:**
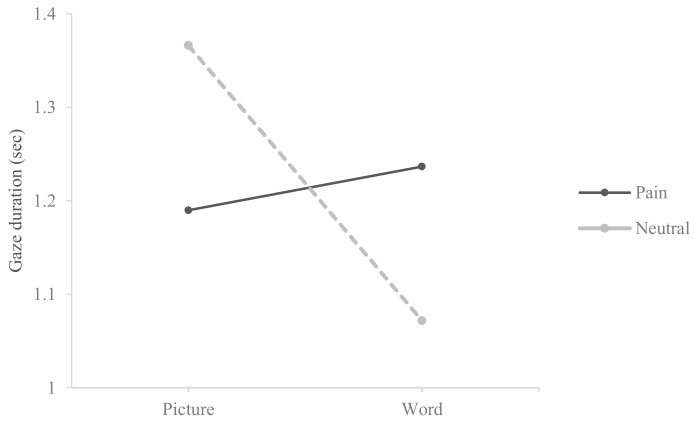
Interaction plot between stimulus type and expression type for pain–neutral pairs.

**Figure 2:**
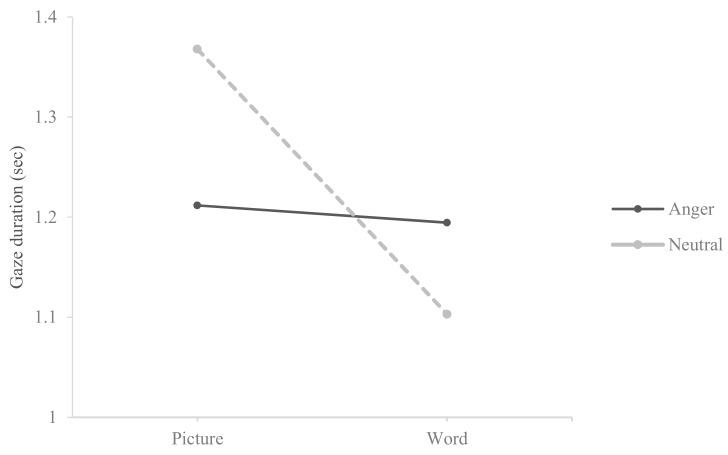
Interaction plot between stimulus type and expression type for anger–neutral pairs.

As shown in Figure 1 and Figure 2, the interaction effects were plotted. These plots showed that chronic pain patients gravitated significantly more toward pain- or anger-related words compared to neutral words while the opposite pattern was true for facial expressions (i.e., participants gravitated significantly more toward neutral facial expressions compared to pain- or anger-related facial expressions).

### 3.3. Time Course of Gaze Durations

Repeated measure ANOVAs with within-factors ‘time’ (0–500 ms vs. 500–1000 ms vs. 1000–1500 ms vs. 1500–2000 ms vs. 2000–2500 ms vs. 2500–3000 ms) and ‘stimulus type’ (pain–neutral vs. anger–neutral) were performed separately for different facial expression and word pairs. As shown in the Table 5, a significant effect was found on time (*F* (5, 185) = 37.058, *p* < 0.001, *η*^2^ = 0.500) for pain and neutral facial expression pairs. Regarding anger–neutral facial expression pairs, a significant effect was found on time (*F* (5, 190) = 38.800, *p* < 0.001, *η*^2^ = 0.505) and interaction between time and stimulus type (*F* (5, 190) = 2.715, *p* < 0.05, *η*^2^ = 0.067). For word stimulus, there were significant effects on time for pain–neutral word pairs (*F* (5, 185) = 13.486, *p* < 0.001, *η*^2^ = 0.267) and for anger–neutral word pairs (*F* (5, 185) = 11.300, *p* < 0.001, *η*^2^ = 0.234).

**Figure 3:**
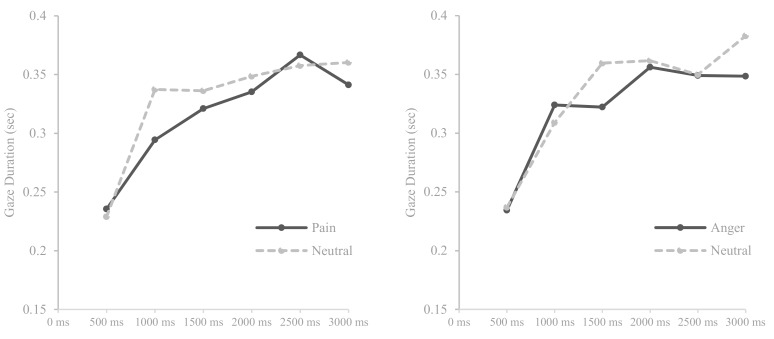
Means of gaze durations for pain and anger faces compared with neutral faces over six times.

**Figure 4:**
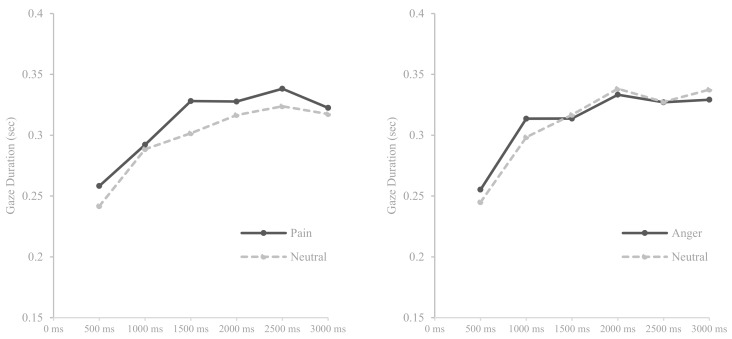
Means of gaze durations for pain and anger-related words compared with neutral words over six times.

Figure 3 shows that participants gazed at facial expressions displaying pain- and anger-related information less than neutral stimuli although participants gazed at anger expressions more than neutral expressions during the initial phase of attention (500–1000 ms). Figure 4 revealed that participants gazed at pain-related words more than neutral words whereas in anger–neutral word pairs, this pattern was not as distinct: From the initial to mid phase of attention (i.e., 0–1500 ms), participants gazed at anger-related words more than neutral words, however, they gazed at both word stimulus in a similar degree from the mid to later stage of attention (i.e., 1500–3000 ms). In general, attentional engagement toward all facial expression stimuli tended to gradually increase during the entire attentional process but for word stimuli, attentional engagement increased from the early stage to middle stage of attention and was maintained during the later stage of attention.

ANOVA was performed with dependent variables: bias scores (pain–neutral word bias score, anger–neutral word bias score, pain–neutral facial expression bias score, anger–anger facial expression bias score) and an independent variable (pain catastrophizing group). Bias scores were calculated by subtracting the total fixation durations of neutral stimuli from the total fixation duration of either pain- or anger-related stimuli. To investigate the role of pain catastrophizing on attentional bias toward pain stimuli, participants were divided into a high pain catastrophizing group (*n* = 21) and a low pain catastrophizing group (*n* = 19) using the median split of scores obtained by pain catastrophizing scale (PCS). As shown in Table 6, significant effects were found on pain–neutral word bias score (*F* (1,38) = 5.183, *p* < 0.05) and anger–neutral word bias score (*F* (1,38) = 4.432, *p* < 0.05). These results indicated that the high pain catastrophizing group gazed significantly more at pain- or anger-related words rather than neutral words compared to the low pain catastrophizing group.

## 4. Discussion

The current study investigated the attentional engagement to pain-related stimuli for different types of pain-related stimuli among chronic pain patients with musculoskeletal disorders. Results revealed attentional preference toward pain-related words when those stimuli were paired with neutral words, which was consistent with previous studies. Previous studies [26,27] discussed attentional inclination toward threat-related information including pain- and anger-related stimuli for healthy individuals and described this attentional pattern as a natural response to threat. In the present study, results also revealed the attentional preference toward anger-related words when those stimuli were paired with neutral words. However, this result did not reach to the level of significance. The discrepancy in gaze durations between threat-related word stimuli and neutral stimuli was greater for pain–neutral linguistic stimuli pairs compared to anger–neutral linguistic stimuli pairs. This result may indicate that chronic pain patients gravitate toward pain-related words because pain word stimuli were relevant to their pain experience and pain-related word stimuli, like negative thoughts in rumination process among depressed patients, might have activated the attentional process where chronic pain patients had difficulty disengaging from pain-related word stimuli. However, follow-up studies should be conducted to support for the pain-specific attentional bias for the word stimuli among chronic pain patients with musculoskeletal disorders.

The first hypothesis of the present study was only partially supported because the attentional pattern observed for facial expression stimuli (i.e., attentional preference toward neutral stimuli) when pain and anger facial expression stimuli were paired with neutral stimuli, was the opposite pattern observed in previous studies [3,28]. This was a puzzling result particularly because our previous studies [3] utilized similar research methods (i.e., same pictorial stimuli and similar experimental design) as the current study. There were few differences between our previous study [3] and the current study. First, in the previous study [3], subjects were young college students who reported various chronic pain issues whereas the subjects in the present study were an older population who were in treatment for their musculoskeletal disorders in the outpatient clinics of the university hospital. Even though seeing photos that contain pained and angry facial expressions on the computer screen is not as anxiety-provoking and threatening as being in situations where individuals anticipate high levels of pain, visual stimuli that evoke immediate responses may increase fear and anxiety of pain more rapidly for chronic pain patients who are in treatment for their chronic pain. Chronic pain patients in the present study may have diverted their attention away from threat-related facial expression stimuli in order to decrease their distress because they struggled more with pain management and experienced higher level of concerns about their pain than young college students who were not in the treatment for their chronic pain in the previous study [3]. However, current results revealed no significant difference between pain and neutral facial expression stimuli, so the evidence for attentional avoidance from pain-related information is inconclusive based on current results. Second, participants in current study had to view both pictorial stimuli and linguistic stimuli for the viewing trials whereas the previous study [3] only had to view pictorial stimuli. Even though the sequence of presenting two types of stimuli was randomized for each participant and the participants had a break between trials, fatigue may have influenced the attentional process of participants (who were older and more physically challenged due to their chronic pain) in the present study.

A previous study [26] argued that presenting pain-related stimuli longer than 2000 ms could provide an opportunity for researchers to observe an attentional pattern to pain-stimuli more clearly, however, studies that examined maintained attention to pain-related information over 2000 ms by utilizing eye tracking methods are lacking. Thus, the present study investigated the time course of attentional engagement toward pain-related information between pictorial and linguistic stimuli across 3000 ms by utilizing an eye tracking method. Consistent with the previous study [3], the attentional engagement toward threat-related information, particularly pain-related information, was maintained across 3000 ms regardless of stimulus type. This result supported the second hypothesis of the present study and provided evidence for the maintained attention toward pain-related information among individuals with musculoskeletal disorders who are in treatment for their chronic pain in the hospital.

The current study showed that higher pain catastrophizing levels are related to greater levels of attentional engagement to pain- and anger-related words. This result may not be consistent with fear-avoidance attentional patterns shown among individuals with chronic pain in previous studies, however, this result may reflect the tendency of chronic pain patient with high levels of pain catastrophizing to ruminate threat-related thoughts. High levels of ruminations for threat-related thoughts may increase attentional engagement to pain- and anger-related words. The maintaining of attention toward pain-related information shown in previous studies is an attentional bias (e.g., rumination process) similar to the negative thoughts observed in depressed individuals [1]. This implies that repetitive negative thoughts related to pain among chronic pain patients with high levels of pain catastrophizing can intensify and maintain the attention of threat-related information. This is an interesting result as the present study only used sensory pain words and excluded emotional pain words for word stimuli, which indicates that high levels of pain catastrophizing may increase attention toward not only word stimuli that suggest threat or negative emotions but also word stimuli that indicate sensory pain.

The same patterns were not observed for the facial expression stimuli. This result was not consistent with previous research suggesting that pain catastrophizing influences pain perceptions by increasing attention toward pain-related facial expression stimuli [10,29]. Either those who were recently diagnosed as chronic pain [10] or those who were suffering from chronic pain but were not seeking treatment (community or college sample) [3,4] may be differently affected by visual stimuli such as facial expressions of pain than those who were experiencing more severe pain and were receiving treatment in a hospital setting for their musculoskeletal disorders. The greater severity, or perhaps the more prolonged experience of pain may cause altered response to pain stimuli compared to community or college samples.

The current research may inform pain psychology therapy by allowing therapists to provide psychoeducation to chronic patients, informing them that their brain may naturally focus more on pain related words. Once patients are made aware, they may be able to alter this automatic reactive response. We can encourage patients to monitor their observation of pain terms and in the future, potentially implement other empirically validated interventions such as cognitive thought stopping, meditation, distraction, mindfulness to non-pain related stimuli, or some new intervention that has yet to be developed to retrain the brain’s response to pain words. Clinicians or health care providers who treat chronic pain patients with musculoskeletal disorders should also be mindful that patients with high levels of pain catastrophizing may experience in difficulty in disengaging from repetitive negative thoughts about pain. Interventions that target to decrease rumination (e.g., behavioral techniques, cognitive restructuring, acceptance and commitment therapy, mindfulness exercises) can be helpful to assist these individuals in decreasing their distress levels and in increasing their capacity to cope with their chronic pain.

Despite the contributions of the present study, there are limitations. First, the study’s limited sample size requires a cautious interpretation of results. However, a power analysis was conducted prior to data collection, and the result indicated that 28 participants were sufficient to address the primary study question. Also, the present study differed from previous studies that investigated attentional bias of non-clinic samples with a variety of chronic pain conditions. The present study focused on chronic pain patients with musculoskeletal disorders in the hospital. Second, chronic pain groups were divided according to their scores of pain catastrophizing, and the statistical method utilized was a median split. Researchers raised concerns about using this method because it can increase type I errors and make it harder to detect possible interaction effects [30]. Recent research has found, however, that the median split is a legitimate statistical method when independent variables are uncorrelated [31]. Lastly, the present study did not include a pain-free control group. Previous studies utilizing eye tracking method either found no attentional bias to pain-related information among healthy individuals [5] or found that healthy individuals reduced their attentional engagement to pain-related stimuli significantly during the later stage of attention [26]. However, future studies may confirm this finding further by including a pain-free control group to compare with a chronic pain group to determine whether the maintained attention toward pain-related information is specific to chronic pain groups.

## 5. Conclusions

The present study expanded previous studies by directly comparing pictorial stimuli (i.e., pained facial expressions) with that of linguistic visual stimuli (i.e., pain-related words) in investigating attentional engagement to pain-related information and the role of pain catastrophizing on attentional engagement to pain-related information. Studies that investigate the time course of maintained attention toward pain-related information on both linguistic and pictorial stimuli were lacking. The current results may underscore the importance of investigating how different modalities of stimuli can differently influence attentional engagement to pain-related information for chronic pain patients. Lastly, consistent with previous studies, the present study supported the role of pain catastrophizing on attentional engagement to pain-related information. The results of the present study can provide useful information for both clinicians and researchers to develop effective psychological interventions for the chronic pain patients with musculoskeletal disorders.

## Figures and Tables

**Table 1:** Descriptive statistics of demographic information, pain-related variables, and psychological variables.

Variables	Mean (SD)
Age	46.58 (16.26)
Gender	
Male	37.5%
Female	62.5%
Smoking	
Yes	20.0%
No	80.0%
Drinking	
Yes	42.5%
No	57.5%
Pain Medication	
Yes	60.0%
No	40.0%
Chronic pain duration (months)	57.51 (79.92)
Current pain intensity (1–10)	5.40 (2.31)
Average pain intensity in past 3 months (1–10)	6.75 (1.86)
Pain days per month	16.42 (7.40)
Pain disability index	38.38 (14.01)
Pain catastrophizing	25.68 (11.26)
Chronic Pain Location	
Arm	10%
Leg	15%
Shoulder	2.5%
Back	50%
Neck	12.5%
Pelvis	5%
Entire body	5%

**Table 2:** Descriptive statistics of demographic information and pain-related variables by two pain catastrophizing scale (PCS) groups.

Variables	Mean (SD)		*F/χ* ^2^
	Low PCS (*n* = 19)	High PCS (*n* = 21)	
Age	42.84 (16.02)	49.95 (16.11)	1.953
Gender			0.327/0.567
Male	42.10%	33.30%	
Female	57.90%	66.70%	
Chronic pain duration (month)	34.79 (38.39)	78.07 (100.97)	3.082
Pain frequency	19.63 (9.34)	17.19 (9.83)	0.645
Average pain intensity in past 3 months	6.53 (2.09)	6.95 (1.66)	0.5158
Pain catastrophizing	16.00 (4.40)	34.43 (7.79)	82.459 ***
Pain disability index	34.84 (12.64)	41.57 (14.71)	2.383

Note: low PCS = low catastrophizing group; high PCS = high catastrophizing group. *** *p* < 0.001.

**Table 3:** Means/SD and *t*-test results of the gaze durations.

	Expression		*t*	*p*	Cohen’s *D*
**ST**	**Pain M (SD)**	**Neutral M (SD)**			
Picture	1.1899 (0.351)	1.3661 (0.417)	−2.017	0.051	0.401
Word	1.2366 (0.447)	1.0718 (0.388)	2.507	0.017 *	0.403
	**Anger M (SD)**	**Neutral M (SD)**	***t***	***p***	
Picture	1.2117 (0.348)	1.3679 (0.397)	−2.128	0.040 *	0.340
Word	1.1945 (0.386)	1.1030 (0.380)	1.222	0.229	0.204

Note: ST = Stimulus type. * *p* < 0.05

**Table 4:** Summary of ANOVA with 2 stimulus type (word vs. picture) × 2 expressions (pain vs. neutral, anger vs. neutral).

Total Gaze (Pain–neutral)	*F*	*p*	*η* ^2^
Stimulus type	3.696	0.056	0.024
Expression type	0.008	0.929	0.000
Stimulus × Expression type	7.018	0.000***	0.044
**Total Gaze (Anger–neutral)**	***F***	***p***	***η*^2^**
Stimulus type	5.427	0.021*	0.034
Expression type	0.286	0.593	0.002
Stimulus × Expression type	4.184	0.043*	0.027

Note: Stimulus type (word vs. picture). Expression type (pain vs. neutral; anger vs, neutral). * *p* < 0.05, *** *p* < 0.001.

**Table 5:** Summary of repeated measure ANOVA for pain–neutral pairs and anger–neutral pairs.

Face (Pain–Neutral)	*F*	*p*	*η* ^2^
Time	37.058	0.000 ***	0.500
Stimulus type	4.079	0.051	0.099
Time × Stimulus type	1.772	0.145	0.046
**Face (Anger–Neutral)**	***F***	***p***	***η*^2^**
Time	38.800	0.000 ***	0.505
Stimulus type	3.444	0.071	0.083
Time × Stimulus type	2.715	0.034*	0.067
**Word (Pain–Neutral)**	***F***	***p***	***η*^2^**
Time	13.486	0.000 ***	0.267
Stimulus type	1.381	0.247	0.036
Time × Stimulus type	0.455	0.757	0.012
**Word (Anger–Neutral)**	***F***	***p***	***η*^2^**
Time	11.300	0.000 ***	0.234
Stimulus type	0.034	0.854	0.001
Time × Stimulus type	0.481	0.754	0.013

Note: * *p* < 0.05, *** *p* < 0.001.

**Table 6:** Summary of ANOVA with pain catastrophizing group as an independent variable, and pain–neutral bias scores and anger–neutral bias scores as dependent variables.

	*BS M (SD)*	*BS Std. Error*	*M (SD)*	*M (SD)*	*F*	p
	Pain–Neutral W	Pain–Neutral W	Pain W	Neutral W		
High CATA	0.3033 (0.5185)	0.12097	1.3585 (0.5377)	1.0552 (0.4808)	5.183	0.029 *
Low CATA	0.0190 (0.1684)	0.03864	1.1083 (0.2869)	1.0893 (0.2692)		
	**Anger–Neutral W**	**Anger–Neutral W**	**Anger W**	**Neutral W**	4.432	0.042 *
High CATA	0.2384 (0.5319)	0.12516	1.3199 (0.4373)	1.0815 (0.4355)		
Low CATA	−0.0633 (0.3359)	0.07705	1.0624 (0.2767)	1.1257 (0.3226)		
	**Pain–Neutral F**	**Pain–Neutral F**	**Pain F**	**Neutral F**		
High CATA	−0.0578 (0.5516)	0.12914	1.2399 (0.4109)	1.2977 (0.4908)	1.983	0.167
Low CATA	−0.3009 (0.5247)	0.12037	1.1372 (0.2749)	1.4381 (0.3188)		
	**Anger–Neutral F**	**Anger–Neutral F**	**Anger F**	**Neutral F**		
High CATA	−0.1169 (0.4204)	0.09898	1.2138 (0.4255)	1.3308 (0.4777)	0.296	0.590
Low CATA	−0.1976 (0.5035)	0.11552	1.2094 (0.2531)	1.4070 (0.2966)		

Note: CATA = catastrophizing; BS = bias Score, W = word stimuli; F = facial expression stimuli. * *p* < 0.05.
